# Crystal structure of (*E*)-1-methyl-2-[2-(2-methoxphen­yl)ethen­yl]-4-nitro-1*H*-imidazole

**DOI:** 10.1107/S1600536814017206

**Published:** 2014-08-01

**Authors:** Hayette Alliouche, Abdelmalek Bouraiou, Sofiane Bouacida, Hocine Merazig, Ali Belfaitah

**Affiliations:** a Laboratoire des Produits Naturels d’Origine Végétale et de Synthèse Organique, PHYSYNOR, Université Constantine 1, 25000 Constantine, Algeria; b Unité de Recherche de Chimie de l’Environnement et Moléculaire Structurale (CHEMS), Université Constantine 1, 25000 , Algeria; c Département Sciences de la Matière, Faculté des Sciences Exactes et Sciences de la Nature et de la Vie, Université Oum El Bouaghi, Algeria

**Keywords:** crystal structure, hydrogen bonding, π–π stacking inter­actions, nitro­imidazoles,

## Abstract

In the asymmetric unit of the title compound, C_13_H_13_N_3_O_3_, the 2-(2-methoxphen­yl)ethenyl unit is connected to the methyl-nitro­imidazole 1-methyl-4-nitro-1*H*-imidazole moiety. The mol­ecule is quasi-planar and the planes of the two rings form a dihedral angle of 0.92 (11)°. The crystal packing can be described as layers parallel to the (011) plane, stabilized by inter­molecular C—H⋯O hydrogen bonding, resulting in the formation of an infinite three-dimensional network linking these layers. Strong π–π stacking inter­actions are observed, *viz.* benzene–benzene, imidazole–imidazole and benzene–imidazole rings, with centroid–centroid distances of 3.528 (2), 3.457 (2) and 3.544 (2) Å, respectively. Intensity statistics indicated twinning by non-merohedry, with refined weighs of the twin components of 0.3687:0.6313.

## Related literature   

For the synthesis and applications of this important class of compounds, see: Hori *et al.* (1997 ▶); Bourdin-Trunz *et al.* (2011 ▶). For our previous work on imidazole derivatives, see: Alliouche *et al.* (2014 ▶); Zama *et al.* (2013 ▶); Bahnous *et al.* (2012 ▶).
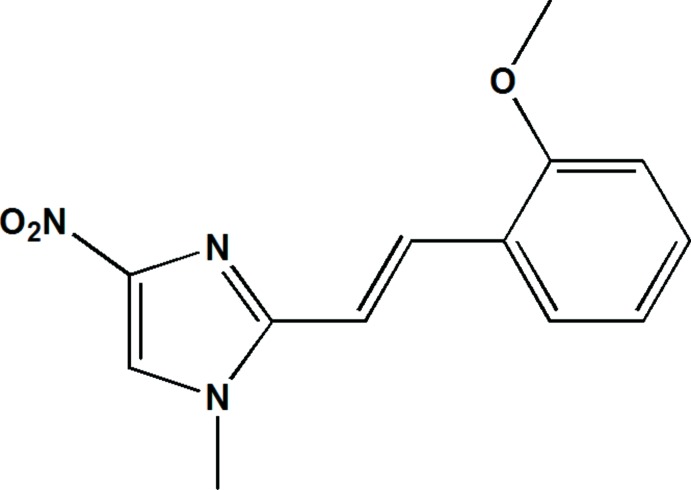


## Experimental   

### Crystal data  

C_13_H_13_N_3_O_3_
*M* *_r_* = 259.26
Triclinic, 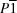

*a* = 7.9339 (18) Å
*b* = 8.1994 (19) Å
*c* = 10.452 (3) Å
α = 68.877 (17)°
β = 75.037 (17)°
γ = 76.182 (17)°
*V* = 604.7 (2) Å^3^
*Z* = 2
Mo *K*α radiation
μ = 0.10 mm^−1^
*T* = 150 K
0.19 × 0.12 × 0.08 mm

### Data collection  

Bruker APEXII diffractometer
Absorption correction: multi-scan (*SADABS*; Sheldrick, 2002 ▶) *T*_min_ = 0.754, *T*_max_ = 1.000
5177 measured reflections
5177 independent reflections
3712 reflections with *I* > 2σ(*I*)

### Refinement  

*R*[*F* ^2^ > 2σ(*F* ^2^)] = 0.088
*wR*(*F* ^2^) = 0.282
*S* = 1.06
5171 reflections
176 parameters
H-atom parameters constrained
Δρ_max_ = 0.49 e Å^−3^
Δρ_min_ = −0.42 e Å^−3^

### 

Data collection: *APEX2* (Bruker, 2006 ▶); cell refinement: *SAINT* (Bruker, 2006 ▶); data reduction: *SAINT*; program(s) used to solve structure: *SIR2002* (Burla *et al.*, 2005 ▶); program(s) used to refine structure: *SHELXL97* (Sheldrick, 2008 ▶); molecular graphics: *ORTEP-3 for Windows* (Farrugia, 2012 ▶) and *DIAMOND* (Brandenburg & Berndt, 2001 ▶); software used to prepare material for publication: *WinGX* (Farrugia, 2012 ▶).

## Supplementary Material

Crystal structure: contains datablock(s) I. DOI: 10.1107/S1600536814017206/hg5400sup1.cif

Structure factors: contains datablock(s) I. DOI: 10.1107/S1600536814017206/hg5400Isup2.hkl

**Fig. 1.**
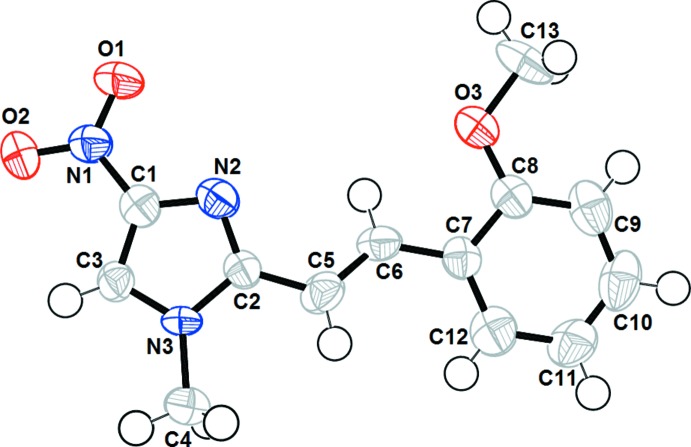
The structure of the title compound with the atomic labelling scheme. Displacement are drawn at the 50% probability level.

**Fig. 2.**
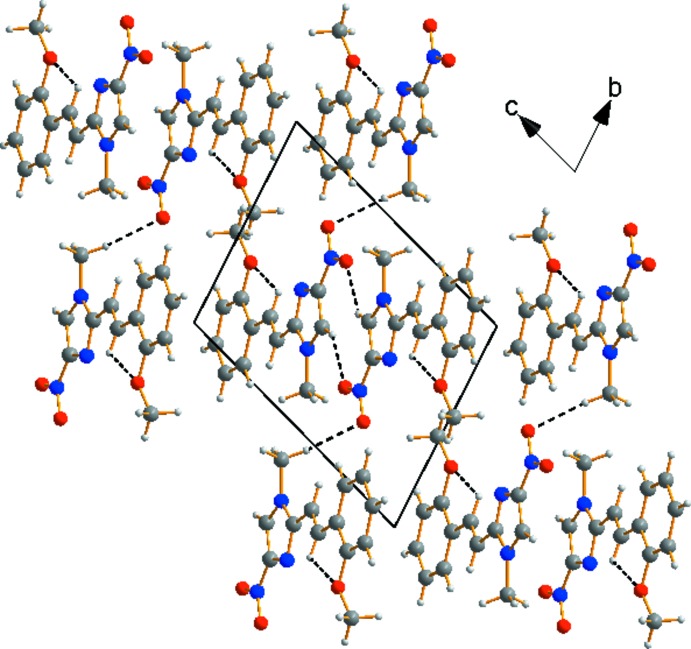
A diagram of the layered crystal packing of (I) viewed down the a axis and showing hydrogen bond [C—H···O] as dashed line.

. DOI: 10.1107/S1600536814017206/hg5400fig1.tif
The structure of the title compound with the atomic labelling scheme. Displacement are drawn at the 50% probability level.

a . DOI: 10.1107/S1600536814017206/hg5400fig2.tif
A diagram of the layered crystal packing of (I) viewed down the *a* axis and showing hydrogen bond [C—H⋯O] as dashed line.

CCDC reference: 1015965

Additional supporting information:  crystallographic information; 3D view; checkCIF report

## Figures and Tables

**Table 1:** Hydrogen-bond geometry (Å, °)

*D*—H⋯*A*	*D*—H	H⋯*A*	*D*⋯*A*	*D*—H⋯*A*
C3—H3⋯O2^i^	0.93	2.45	3.271 (4)	147
C4—H4*B*⋯O1^ii^	0.96	2.53	3.465 (5)	165
C6—H6⋯O3	0.93	2.31	2.685 (4)	103
C6—H6⋯N2	0.93	2.60	2.935 (4)	102

Symmetry codes: (i) 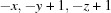; (ii) 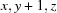.

## supplementary crystallographic information

### S1. Comment

Nitroimidazoles are a class of N-heterocyclic compounds which have a wide range of applications in the drug synthesis (Hori, *et al.*, 1997) In fact, Metronidazole (Flagyl) and related N-1 substituted 5-nitroimidazoles such as Tinidazole (Fasigyne), Ornidazole (Tiberal) and Secnidazole (Secnol) still commonly used in medicine. Despite their fewer biological activities compared with 5-nitroimidazoles, a number of 4-nitroimidazoles were reported to exhibit antileishmanial, antiamebic and anti-parasitic activities (Bourdin-Trunz, *et al.* 2011). However, only some limited investigations have been carried out using methyl iodide (Alliouche, *et al.* 2014). In previous work, we have reported the synthesis and structure determination of some new heterocyclic compounds bearing an imidazole unit (Zama, *et al.*, 2013; Bahnous, *et al.*, 2012). Herein, we describe the synthesis and the structure determination of (*E*)-1-methyl-2-[(2-methoxphenyl)-1-ethenyl]-4-nitroimidazole resulting from the intramolecular transposition reaction of its 5-nitro isomer in the presence of catalytic amounts of methyl iodide in nitrobenzene. The molecular geometry and the atom-numbering scheme of (I) are shown in Fig. 1. In the asymmetric unit of title compound the methoxphenyl-1-ethenyl unit is linked to methyl-nitroimidazole moiety. The molecule is quasi-planar and the two rings of phenyl and imidazol form a dihedral angle of 0.92 (11)°. The crystal packing can be described as layers parallel to (011) plane, along the *a* axis (Fig. 2). It is stabilized by intermolecular hydrogen bond C—H···O, resulting in the formation of an infinite three-dimensional network linking these layers together and reinforcing cohesion in the structure (Fig. 2). Hydrogen-bonding parameters are listed in Table 1. Strong π-π stacking interactions are observed between phenyl-phenyl, imidazol-imidazol and phenyl-imidazol rings, distances centroid-centroid are *Cg*—*Cg* = 3.528 (2), 3.457 (2) and 3.544 (2) Å respetively. The crystal used was a non-merohedral twin, the refined ratio of twin components being 0.3687:0.6313.

### S2. Experimental

The title compound was obtained as a yellow-green solid in 83% of yield by heating a solution of (*E*)-1-methyl-2-[(2-methoxphenyl)-1-ethenyl]-5-nitroimidazole in nitrobenzene at 160°C during 24 h in the presence of catalytic amount of CH_3_I. Suitable crystal of compound (I) was obtained by slow evaporation from a water/methanol solution at room temperature, and X-ray crystallographic analysis confirmed the structural assignment (Fig. 1).

### S3. Refinement

All non-H atoms were refined with anisotropic atomic displacement parameters. All H atoms were localized on Fourier maps but introduced in calculated positions and treated as riding on their parent C or N atom. (with C—H = 0.93 and 0.96 Å and *U*_iso_(H) = 1.5 or 1.2 (carrier atom).

### Crystal data

**Table:**

C_13_H_13_N_3_O_3_	*Z* = 2
*M**_r_* = 259.26	*F*(000) = 272
Triclinic, *P*1	*D*_x_ = 1.424 Mg m^−^^3^
*a* = 7.9339 (18) Å	Mo *K*α radiation, λ = 0.71073 Å
*b* = 8.1994 (19) Å	Cell parameters from 1356 reflections
*c* = 10.452 (3) Å	θ = 2.7–24.6°
α = 68.877 (17)°	µ = 0.10 mm^−^^1^
β = 75.037 (17)°	*T* = 150 K
γ = 76.182 (17)°	Block, yellow
*V* = 604.7 (2) Å^3^	0.19 × 0.12 × 0.08 mm

### Data collection

**Table:**

Bruker APEXII diffractometer	3712 reflections with *I* > 2σ(*I*)
Graphite monochromator	*R*_int_ = 0
CCD rotation images, thin slices scans	θ_max_ = 25.3°, θ_min_ = 2.7°
Absorption correction: multi-scan (*SADABS*; Sheldrick, 2002)	*h* = −9→9
*T*_min_ = 0.754, *T*_max_ = 1.000	*k* = −9→9
5177 measured reflections	*l* = −12→12
5177 independent reflections	

### Refinement

**Table:**

Refinement on *F*^2^	Secondary atom site location: difference Fourier map
Least-squares matrix: full	Hydrogen site location: inferred from neighbouring sites
*R*[*F*^2^ > 2σ(*F*^2^)] = 0.088	H-atom parameters constrained
*wR*(*F*^2^) = 0.282	*w* = 1/[σ^2^(*F*_o_^2^) + (0.1745*P*)^2^ + 0.1919*P*] where *P* = (*F*_o_^2^ + 2*F*_c_^2^)/3
*S* = 1.06	(Δ/σ)_max_ < 0.001
5171 reflections	Δρ_max_ = 0.49 e Å^−^^3^
176 parameters	Δρ_min_ = −0.42 e Å^−^^3^
0 restraints	Extinction correction: *SHELXL97* (Sheldrick, 2008), Fc^*^=kFc[1+0.001xFc^2^λ^3^/sin(2θ)]^-1/4^
Primary atom site location: structure-invariant direct methods	Extinction coefficient: 0.045 (12)

### Special details

**Table:**

Geometry. All e.s.d.'s (except the e.s.d. in the dihedral angle between two l.s. planes) are estimated using the full covariance matrix. The cell e.s.d.'s are taken into account individually in the estimation of e.s.d.'s in distances, angles and torsion angles; correlations between e.s.d.'s in cell parameters are only used when they are defined by crystal symmetry. An approximate (isotropic) treatment of cell e.s.d.'s is used for estimating e.s.d.'s involving l.s. planes.
Refinement. Refinement of *F*^2^ against ALL reflections. The weighted *R*-factor *wR* and goodness of fit *S* are based on *F*^2^, conventional *R*-factors *R* are based on *F*, with *F* set to zero for negative *F*^2^. The threshold expression of *F*^2^ > σ(*F*^2^) is used only for calculating *R*-factors(gt) *etc*. and is not relevant to the choice of reflections for refinement. *R*-factors based on *F*^2^ are statistically about twice as large as those based on *F*, and *R*- factors based on ALL data will be even larger.

### Fractional atomic coordinates and isotropic or equivalent isotropic displacement parameters (Å^2^)

**Table:**

	*x*	*y*	*z*	*U*_iso_*/*U*_eq_	
O1	0.4178 (3)	0.2400 (3)	0.2786 (3)	0.0256 (6)	
O3	1.0969 (3)	0.6148 (3)	0.0846 (2)	0.0219 (6)	
O2	0.1531 (3)	0.2922 (3)	0.3961 (3)	0.0238 (6)	
N1	0.3026 (4)	0.3303 (4)	0.3420 (3)	0.0167 (7)	
N2	0.5059 (3)	0.5331 (4)	0.2979 (3)	0.0162 (7)	
N3	0.3292 (3)	0.7076 (3)	0.4156 (3)	0.0134 (6)	
C1	0.3460 (4)	0.4809 (4)	0.3545 (3)	0.0142 (7)	
C2	0.4934 (4)	0.6726 (4)	0.3362 (3)	0.0125 (7)	
C5	0.6299 (4)	0.7796 (5)	0.3004 (3)	0.0172 (8)	
H5	0.6018	0.8867	0.319	0.021*	
C6	0.7973 (4)	0.7279 (4)	0.2408 (3)	0.0151 (7)	
H6	0.8198	0.6187	0.2261	0.018*	
C7	0.9464 (4)	0.8227 (4)	0.1968 (3)	0.0139 (7)	
C8	1.1027 (4)	0.7634 (4)	0.1144 (3)	0.0159 (8)	
C13	1.2438 (4)	0.5580 (5)	−0.0106 (4)	0.0273 (9)	
H13C	1.2622	0.6542	−0.0961	0.041*	
H13A	1.22	0.4604	−0.0296	0.041*	
H13B	1.3478	0.5214	0.0299	0.041*	
C4	0.2711 (4)	0.8439 (5)	0.4845 (3)	0.0196 (8)	
H4A	0.1465	0.8508	0.5215	0.029*	
H4B	0.2948	0.9563	0.418	0.029*	
H4C	0.3339	0.814	0.5591	0.029*	
C3	0.2328 (4)	0.5873 (4)	0.4277 (3)	0.0139 (7)	
H3	0.117	0.5778	0.4744	0.017*	
C9	1.2469 (4)	0.8506 (5)	0.0702 (3)	0.0207 (8)	
H9	1.349	0.81	0.0154	0.025*	
C10	1.2392 (5)	0.9970 (5)	0.1072 (3)	0.0233 (8)	
H10	1.3369	1.0544	0.0781	0.028*	
C11	1.0871 (4)	1.0600 (5)	0.1876 (3)	0.0211 (8)	
H11	1.0818	1.1602	0.2116	0.025*	
C12	0.9434 (4)	0.9723 (5)	0.2317 (3)	0.0199 (8)	
H12	0.8418	1.0144	0.2863	0.024*	

### Atomic displacement parameters (Å^2^)

**Table:**

	*U*^11^	*U*^22^	*U*^33^	*U*^12^	*U*^13^	*U*^23^
O1	0.0232 (13)	0.0238 (14)	0.0331 (14)	−0.0046 (11)	0.0055 (11)	−0.0199 (12)
O3	0.0169 (13)	0.0238 (14)	0.0257 (13)	−0.0036 (10)	0.0029 (10)	−0.0135 (11)
O2	0.0142 (13)	0.0280 (15)	0.0313 (14)	−0.0066 (11)	0.0037 (11)	−0.0154 (12)
N1	0.0173 (15)	0.0150 (15)	0.0183 (14)	−0.0021 (12)	−0.0013 (12)	−0.0077 (12)
N2	0.0144 (14)	0.0181 (15)	0.0159 (14)	−0.0007 (12)	−0.0022 (11)	−0.0070 (12)
N3	0.0139 (14)	0.0113 (14)	0.0162 (13)	−0.0006 (11)	−0.0005 (11)	−0.0083 (11)
C1	0.0135 (16)	0.0166 (17)	0.0122 (16)	−0.0007 (13)	−0.0053 (13)	−0.0032 (13)
C2	0.0120 (16)	0.0162 (17)	0.0099 (14)	−0.0020 (13)	−0.0029 (13)	−0.0043 (13)
C5	0.0170 (18)	0.0181 (17)	0.0207 (17)	−0.0027 (14)	−0.0072 (14)	−0.0086 (15)
C6	0.0155 (17)	0.0144 (17)	0.0177 (16)	0.0010 (14)	−0.0055 (13)	−0.0084 (14)
C7	0.0154 (17)	0.0165 (17)	0.0089 (15)	−0.0037 (14)	−0.0028 (13)	−0.0018 (13)
C8	0.0143 (17)	0.0193 (18)	0.0134 (16)	−0.0016 (14)	−0.0034 (14)	−0.0047 (14)
C13	0.0200 (19)	0.033 (2)	0.0253 (19)	0.0037 (17)	0.0043 (15)	−0.0167 (17)
C4	0.0171 (17)	0.0219 (18)	0.0232 (17)	−0.0021 (15)	−0.0010 (14)	−0.0137 (15)
C3	0.0126 (17)	0.0178 (18)	0.0129 (15)	−0.0037 (14)	−0.0026 (13)	−0.0058 (13)
C9	0.0150 (17)	0.029 (2)	0.0163 (17)	−0.0043 (15)	−0.0043 (14)	−0.0036 (15)
C10	0.026 (2)	0.030 (2)	0.0173 (17)	−0.0148 (16)	−0.0081 (15)	−0.0020 (16)
C11	0.0237 (19)	0.025 (2)	0.0201 (17)	−0.0062 (16)	−0.0086 (15)	−0.0092 (16)
C12	0.0187 (18)	0.025 (2)	0.0176 (17)	−0.0039 (15)	−0.0067 (14)	−0.0068 (15)

### Geometric parameters (Å, º)

**Table:**

O1—N1	1.241 (3)	C7—C8	1.406 (4)
O3—C8	1.375 (4)	C8—C9	1.383 (5)
O3—C13	1.429 (4)	C13—H13C	0.96
O2—N1	1.236 (3)	C13—H13A	0.96
N1—C1	1.415 (4)	C13—H13B	0.96
N2—C2	1.317 (4)	C4—H4A	0.96
N2—C1	1.354 (4)	C4—H4B	0.96
N3—C3	1.339 (4)	C4—H4C	0.96
N3—C2	1.380 (4)	C3—H3	0.93
N3—C4	1.464 (4)	C9—C10	1.372 (5)
C1—C3	1.380 (4)	C9—H9	0.93
C2—C5	1.445 (5)	C10—C11	1.386 (5)
C5—C6	1.351 (5)	C10—H10	0.93
C5—H5	0.93	C11—C12	1.382 (5)
C6—C7	1.455 (5)	C11—H11	0.93
C6—H6	0.93	C12—H12	0.93
C7—C12	1.394 (5)		
			
C8—O3—C13	117.1 (3)	O3—C13—H13A	109.5
O2—N1—O1	122.5 (3)	H13C—C13—H13A	109.5
O2—N1—C1	119.0 (3)	O3—C13—H13B	109.5
O1—N1—C1	118.4 (3)	H13C—C13—H13B	109.5
C2—N2—C1	103.9 (3)	H13A—C13—H13B	109.5
C3—N3—C2	108.4 (2)	N3—C4—H4A	109.5
C3—N3—C4	124.7 (3)	N3—C4—H4B	109.5
C2—N3—C4	126.7 (3)	H4A—C4—H4B	109.5
N2—C1—C3	112.9 (3)	N3—C4—H4C	109.5
N2—C1—N1	123.0 (3)	H4A—C4—H4C	109.5
C3—C1—N1	124.1 (3)	H4B—C4—H4C	109.5
N2—C2—N3	111.1 (3)	N3—C3—C1	103.7 (3)
N2—C2—C5	125.9 (3)	N3—C3—H3	128.1
N3—C2—C5	123.0 (3)	C1—C3—H3	128.1
C6—C5—C2	121.7 (3)	C10—C9—C8	120.0 (3)
C6—C5—H5	119.2	C10—C9—H9	120
C2—C5—H5	119.2	C8—C9—H9	120
C5—C6—C7	127.7 (3)	C9—C10—C11	120.6 (3)
C5—C6—H6	116.1	C9—C10—H10	119.7
C7—C6—H6	116.1	C11—C10—H10	119.7
C12—C7—C8	117.2 (3)	C12—C11—C10	119.2 (3)
C12—C7—C6	123.3 (3)	C12—C11—H11	120.4
C8—C7—C6	119.5 (3)	C10—C11—H11	120.4
O3—C8—C9	124.8 (3)	C11—C12—C7	121.9 (3)
O3—C8—C7	114.1 (3)	C11—C12—H12	119
C9—C8—C7	121.1 (3)	C7—C12—H12	119
O3—C13—H13C	109.5		
			
C2—N2—C1—C3	0.3 (3)	C13—O3—C8—C9	−6.8 (5)
C2—N2—C1—N1	−178.3 (3)	C13—O3—C8—C7	173.6 (3)
O2—N1—C1—N2	−179.5 (3)	C12—C7—C8—O3	179.6 (3)
O1—N1—C1—N2	1.5 (4)	C6—C7—C8—O3	−0.6 (4)
O2—N1—C1—C3	2.0 (5)	C12—C7—C8—C9	−0.1 (5)
O1—N1—C1—C3	−176.9 (3)	C6—C7—C8—C9	179.8 (3)
C1—N2—C2—N3	0.1 (3)	C2—N3—C3—C1	0.6 (3)
C1—N2—C2—C5	−179.2 (3)	C4—N3—C3—C1	−175.4 (3)
C3—N3—C2—N2	−0.5 (3)	N2—C1—C3—N3	−0.5 (3)
C4—N3—C2—N2	175.4 (3)	N1—C1—C3—N3	178.1 (3)
C3—N3—C2—C5	178.9 (3)	O3—C8—C9—C10	−179.3 (3)
C4—N3—C2—C5	−5.3 (5)	C7—C8—C9—C10	0.3 (5)
N2—C2—C5—C6	−10.8 (5)	C8—C9—C10—C11	−0.7 (5)
N3—C2—C5—C6	170.0 (3)	C9—C10—C11—C12	0.8 (5)
C2—C5—C6—C7	178.6 (3)	C10—C11—C12—C7	−0.5 (5)
C5—C6—C7—C12	11.0 (5)	C8—C7—C12—C11	0.1 (5)
C5—C6—C7—C8	−168.8 (3)	C6—C7—C12—C11	−179.7 (3)

### Hydrogen-bond geometry (Å, º)

**Table:**

*D*—H···*A*	*D*—H	H···*A*	*D*···*A*	*D*—H···*A*
C3—H3···O2^i^	0.93	2.45	3.271 (4)	147
C4—H4*B*···O1^ii^	0.96	2.53	3.465 (5)	165
C6—H6···O3	0.93	2.31	2.685 (4)	103
C6—H6···N2	0.93	2.60	2.935 (4)	102

Symmetry codes: (i) −*x*, −*y*+1, −*z*+1; (ii) *x*, *y*+1, *z*.
