# Supplementary figures and images for: Crystal structure of (E)-1-methyl-2-[2-(2-methoxphen­yl)ethen­yl]-4-nitro-1H-imidazole
Source: Acta Crystallogr Sect E Struct Rep Online. 2014 Aug 1;70(Pt 9):o966–7. doi: 10.1107/S1600536814017206 (PMC4186069; doi:10.1107/S1600536814017206)

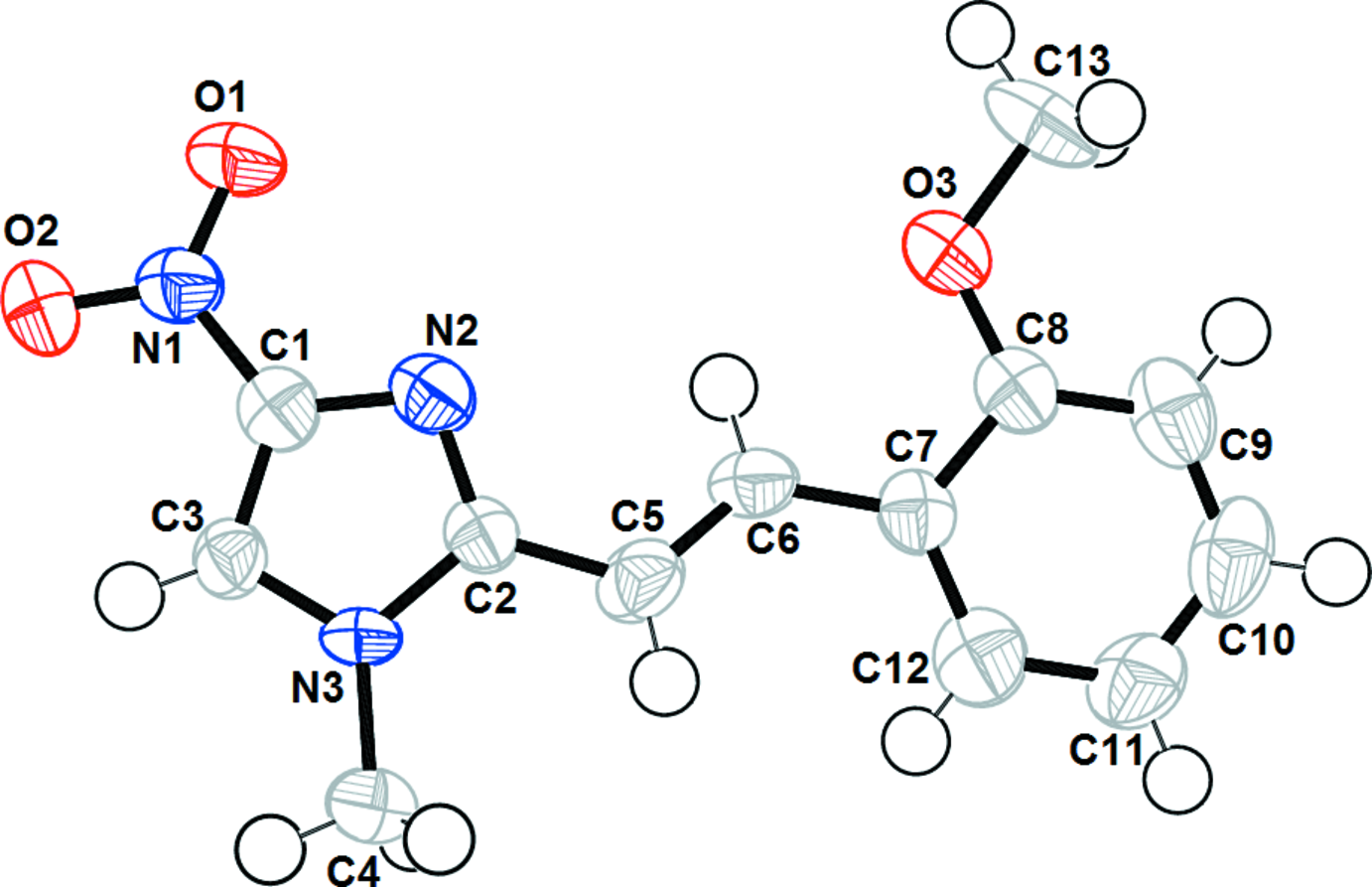

Supplement: Supplementary file 3 [file e-70-0o966-fig1.tif]

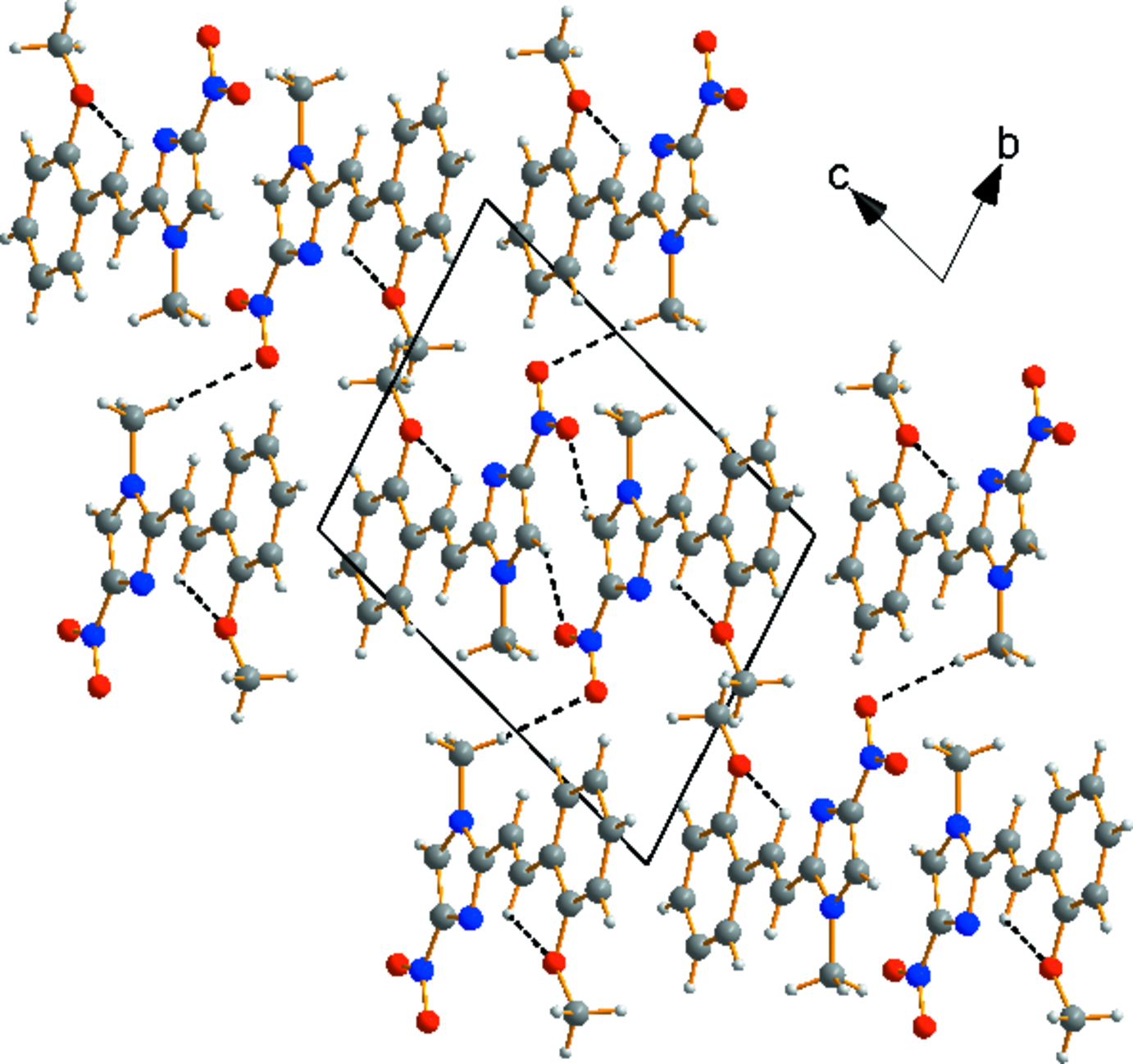

Supplement: Supplementary file 4 [file e-70-0o966-fig2.tif]
